# *Euterpe oleracea* Extract (Açaí) Is a Promising Novel Pharmacological Therapeutic Treatment for Experimental Endometriosis

**DOI:** 10.1371/journal.pone.0166059

**Published:** 2016-11-16

**Authors:** Daniel Escorsim Machado, Karina Cristina Rodrigues-Baptista, Jessica Alessandra-Perini, Roberto Soares de Moura, Thiago Alves dos Santos, Kariny Gomes Pereira, Yasmin Marinho da Silva, Pergentino José Cunha Souza, Luiz Eurico Nasciutti, Jamila Alessandra Perini

**Affiliations:** 1 Unidade de Farmácia, Centro Universitário Estadual da Zona Oeste, Rio de Janeiro, RJ, Brasil; 2 Programa de Pós-Graduação em Saúde Pública e Meio Ambiente, Escola Nacional de Saúde Pública, Fundação Osvaldo Cruz, Rio de Janeiro, RJ, Brasil; 3 Departamento de Farmacologia e Psicobiologia, Universidade Estadual do Rio de Janeiro, Rio de Janeiro, RJ, Brasil; 4 Programa de Pós-Graduação em Biociências e Biotecnologia, Universidade Estadual do Norte Fluminense, Campos dos Goytacazes, RJ, Brasil; 5 Departamento de Farmácia, Universidade Federal do Pará, Belém, PA, Brasil; 6 Instituto de Ciências Biomédicas, Universidade Federal do Rio de Janeiro, Rio de Janeiro, RJ, Brasil; West China Second Hospital, Sichuan University, CHINA

## Abstract

This study investigated the therapeutic potential of *Euterpe oleracea* extract (açaí) on the growth and survival of endometriotic lesions using an experimental model. Twenty female Sprague-Dawley rats were randomized into two groups after the implantation and establishment of autologous endometrium onto the peritoneum abdominal wall and treated with 200 mg/kg hydroalcoholic solution extract from açaí stone or vehicle via gastric tube for 30 consecutive days. Body weight, lesion surface areas, histological and immunohistochemistry analyses of vascular endothelial growth factor (VEGF), VEGF receptor-2 (VEGFR-2), metalloproteinase-9 (MMP-9), cyclooxygenase-2 (COX-2) and F4-80 were performed. Levels of VEGF, VEGFR-2, MMP-9 and COX-2 mRNA were measured. Flow cytometry of F4-80 was performed, and ELISA immunoassays measured prostaglandin E_2_ (PGE_2_), VEGF and nitric oxide (NO) and concentrations. Macrophage cell line J774.G8 was treated with 10, 20, and 40 μg/mL of açaí for 24, 48 and 72 h, and cell viability was measured using 3-(4,5-dimethylthiazol-2-yl)-2,5-diphenyltetrazolium bromide (MTT) assays. Açaí treatment significantly decreased the implant size, and histological examination indicated atrophy and regression. A reduction in immunostaining and mRNA expression of VEGF, MMP-9 and COX-2 was observed, and F4-80 was lower in the treated group than the control group. The treated group also exhibited lower concentrations of PGE_2_, VEGF and NO compared to the control group. Macrophages cells treated with 20 and 40 μg/ml of açaí reduced cell viability in about 50% after 24, 48 and 72 h. Our results suggest that açaí effectively suppressed the establishment and growth of endometriotic lesions, and this agent is a promising novel pharmacological therapeutic treatment for endometriosis.

## Introduction

Endometriosis is defined as the presence of functional endometrium outside the uterine cavity that consists of proliferating functional endometrial glands and stroma [1]. It is an inflammatory disease associated with chronic pelvic pain and infertility, and it results in a markedly reduced quality of life [2]. The prevalence of the disease is in 5–10% of women of reproductive age [3].

The exact pathogenic mechanisms of endometriosis are not known, but several studies demonstrated that the development of a new vascular supply was essential for the establishment and growth of endometriotic lesions [4–6]. Various lines of evidence indicate that growth factors, including vascular endothelial growth factor (VEGF), cytokines and prostaglandins promote the development of endometriosis [7–9]. Notably, endometriotic lesions exhibit increased cyclooxygenase-2 (COX-2) expression compared to eutopic endometrium [10]. COX-2 and VEGF studies are associated with endometriosis and reinforce the hypothesis that the angiogenesis process and inflammation are crucial to the pathophysiology of this disease [8,11,12].

The current treatment for endometriosis is medical and/or surgical. Hormone therapy is the commonly used medical treatment, and it involves oral contraceptives, progestogens and gonadotropin-releasing hormone agonists, which induce a hypoestrogenic state [13]. However, these therapies can only be prescribed for a short time because of serious adverse effects, such as pseudomenopause, massive hemorrhage and bone density loss. The disease recurs within 3–5 years in 30–50% of women after surgical removal of endometriosis lesions [14]. Therefore, the search for additional strategies to effectively treat endometriosis is fundamental.

*Euterpe oleracea* Mart. (Arecaceae), popularly known as “açaí”, is an economically important plant that is found widely in the Amazon region of Brazil. Chemical studies demonstrated that açaí exhibits a diverse composition of hydroxybenzoic acids, antioxidant polyphenolics, flavan-3-ols, and anthocyanins, predominantly cyanidin 3-O-rutinoside and cyanidin 3-O-glucuronide [15–19]. Açaí exhibits antioxidant, antinociceptive, anti-inflammatory and anticancer activities because of its high level of phytochemicals [20–26]. Açaí exhibits exceptional activity against superoxides, inhibits reactive oxygen species (ROS) formation and may inhibit COX-2 [27]. A significant antinociceptive effect of açaí was observed in a spinal nerve ligation model in rats, which suggests the possible development of a new analgesic drug [26]. Recently, Marques *et al*. conducted cytogenetic tests with three doses of açaí in rat cells, and showed that açaí had no significant genotoxic effects in the analyzed cells [28].

Endometriosis is an inflammatory disease and açaí extract may be an effective treatment strategy. The present study investigated the pharmacological effects of açaí on the establishment and growth of endometriotic lesions using an experimental model. We used several assays to investigate anti-inflammatory functions, including an activated macrophage study and nitric oxide (NO) assay. We also investigated whether açaí modulated the angiogenesis process to better understand the mechanisms of action of the extract in the development of endometriosis.

## Materials and Methods

### Preparation of açaí extract

Açaí fruits were obtained from the Amazon Bay (Belém do Pará, Pará, Brazil), excicata number 29052 Museu Goeldi–Belém do Pará, and these study has been authorized by Conselho Nacional de Desenvolvimento Científico e Tecnológico—CNPq (Authorization: 010564/2015-2). We used in our study the hydroalcoholic solution extracted from açaí stones, as previously described [22,23,26]. Briefly, 200 g of açaí stone were boiled in 400 ml of distilled water for 10 min with mixed for 2 min. The decoction was cooled to room temperature and extracted by addition of 400 ml of ethanol with shaking for 2 h. The hydroalcoholic extract was stored (4°C) for 10 days and filtered through Whatman filter paper. Ethanol was evaporated under low pressure at 55°C. The extract was lyophilized (Fisatom Equipamentos Científicos Ltda São Paulo) at temperatures from -30 to -40°C and under a vacuum of 200 mmHg, and frozen at -20°C.

### Experimental animal model

Animals were treated in accordance with protocols approved by the State University of West Zone (UEZO) Institutional Animal Care and Use Committee (CEUA), protocol code CEUA-UEZO-002/2013, and all experiments were conducted in accordance with the ethical guidelines from the CEUA and the NIH Guidelines for the Care and Use of Laboratory Animals (http://oacu.od.nih.gov/regs/index.htm. 8th Edition; 2011).

Twenty female Sprague-Dawley rats (250–300 g) were used after reaching maturity at 8 weeks of age and were housed in polyethylene cages in the Bioterium of UEZO, and were kept at a constant temperature (25°C) under a 12-h light/dark cycle with free access to food and water.

With use of the method described by Vernon and Wilson [29], the animals were anesthetized with intramuscular injection of ketamine and xylazine. The abdomen was opened through a 3-cm midline incision to expose the uterus. One uterine horn was removed and the segment was placed in phosphate-buffered saline at 37°C and split longitudinally, 5×5mm pieces were sectioned and anchored with the endometrium side onto the peritoneum of the ventral abdominal wall by nonadsorbable polypropylene sutures (6–0 Prolene, Ethicon, Piscataway, NJ). Then, the abdomen was closed and the animals were allowed to recover from anesthesia.

### Animal Treatment

Two weeks after the initial implant, ventral midline laparotomy was performed to determine the attachment and viability of endometrial explants. The animals were divided randomly into two groups of each ten animals: açaí group was treated with daily 200 mg/kg body weight, dissolved in saline, by gastric tube for 30 consecutive days, and control group received saline as vehicle by gastric tube for 30 consecutive days. Body weight was measured every three day. After treatment, the rats were euthanized by anesthesia overdose, the peritoneal fluid was collected to flow cytometry, ELISA immunoassay and NO dosage, and the surface areas of the explant (length x width) were evaluated using ImageJ software (National Institutes of Health, Bethesda, MN). Each sample was dissected and immediately divided into one piece that was fixed in 10% buffered formalin and paraffin embedded for histologic and immunohistochemical studies, and another piece that was frozen in liquid nitrogen for RNA extraction.

### Histology and Immunohistochemistry

Formalin-fixed tissues were paraffin-embedded and cut into 4-micrometers-thick sections. Part of the sections were stained with Harris hematoxylin and eosin (HE), and examined microscopically at 200× magnification for the presence of histological hallmarks of endometriosis, such as endometrial glands and stroma. The other paraffin-embedded tissue sections were placed on silane-treated slides, and maintained at room temperature, as previously described [30]. Sections were incubated with the following antibodies: monoclonal antibody against VEGF SC-57496 (Santa Cruz Biotechnology, Santa Cruz, CA) at 1:100 dilution, monoclonal antibody against Flk-1 SC-6251 (Santa Cruz Biotechnology, Santa Cruz, CA) at 1:100 dilution, polyclonal antibody against metalloproteinase-9 (MMP-9) SC-6840 (Santa Cruz Biotechnology, Santa Cruz, CA) at 1:200 dilution, polyclonal antibody against COX-2 SC-1747 (Santa Cruz Biotechnology, Santa Cruz, CA) at 1:100 dilution, and monoclonal antibody against F4-80 macrophage antigen SC-26642 (Santa Cruz Biotechnology, Santa Cruz, CA) at 1:200 dilution. Incubations were carried out overnight and then revealed using LSAB2 Kit HRP, rat (Dako-Cytomation, Carpinteria, CA) with diaminobenzidine (3,3’-diaminobenzidine tablets; Sigma, St. Louis, MO) as the chromogen and counterstained with hematoxylin. For each case, negative control slides consisted of sections incubated with antibody vehicle or no immune rabbit or mouse serum.

### Morphometric analysis

All tissues were examined by two blinded observers using a 400× magnification on light microscope (Nikon, Tokyo, Japan) connected to a digital camera (Coolpix 990; Nikon). Ten fields of an immunostained section (VEGF, VEGFR-2, MMP-9, COX-2 and F4-80) were chosen at random and captured from each specimen. Quantification was assessed on captured highquality images (2048 × 1536 pixels buffer) using the Image Pro Plus 4.5.1 (Media Cybernetics, Silver Spring, MD). Data were stored in Adobe Photoshop, version 3.0, to enable uneven illumination and background color to be corrected. Histologic scores (H) for VEGF, VEGFR-2, MMP-9, COX-2 and F4-80 were calculated using the formula H = ΣPi, where I is the intensity ranging from 0 (negative cells) to 3 (deeply staining cells) and P is the percentage of staining cells for each given i, with P values of 1, 2, 3, 4, and 5 indicating <15%, 15–50%, 50–85%, >85%, and 100% positive-staining cells, respectively. The staining result was expressed as mean ± standard deviations.

### TaqMan real-time reverse transcription-polymerase chain reaction

The m-RNA levels were quantified by TaqMan real-time polymerase chain reaction. RNA from endometriosis samples was isolated using the Trizol^®^ reagent according to the manufacturer’s instructions, and quantified by the Nanodrop^®^ spectrophotometer. Two micrograms of total RNA was used as a template for cDNA synthesis, using the SuperScript II^®^ reverse transcriptase kit (Invitrogen^®^). TaqMan Universal PCR Master Mix (Applied Biosystems^®^) and a validated TaqMan assay was purchased from Applied Biosystems, and were used to quantify mouse *VEGF* (Mm01281449_m1), kinase insert domain receptor (*KDR*), gene which encodes VEGFR-2 (Mm01222421_m1), *MMP-9* (Mm004422991_m1) and *PTGS*, gene which encodes COX-2 (Mm00478374_m1) expression levels, with glyceraldehyde-3-phosphate dehydrogenase (*GAPDH*) (Mm99999915_g1) as an endogenous control. Triplicate TaqMan PCR assays for each gene target were performed in cDNA samples. Real-time reactions were conducted in a 7500 Real-Time thermocycler (Applied Biosystems^®^). The relative quantification of the target genes was performed using the Delta-Delta Ct method.

### ELISA Immunoassay

Peritoneal fluid was collected by rinsing the abdominal cavity with 10 mL of PBS and immediately centrifuged at 1500 rpm during 10 min. Supernatants were stored at -70°C until assayed for VEGF and PGE_2_ by use of an enzyme immunoassay kit (Boster Biological Technology, Pleasanton, CA and Cayman Chemical, Ann Arbor, MI), according to manufacturer's instructions. The concentrations were calculated from standard curves and all samples were assessed in triplicate. The VEGF and PGE_2_ measurement were performed by an automatic plate reader (Spectra Max; Molecular Devices, Sunnyvale, Calif) controlled by SoftMax software (Molecular Devices).

### Flow cytometry

Peritoneal fluid was obtained ex vivo from rat after the treatments by washing twice with PBS, pH 7.2 containing 3% Fetal Bovine Serum (FBS) for flow cytometry analysis. For the control group, the same procedure was conducted. The cells were incubated with Fc blocking (clone 2.4G2) for 10 min. Before, the cells were incubated with monoclonal antibody FITC anti-F4/-80 (BD Biosciences, USA). Samples were analyzed on a flow cytometer (FACSCalibur, BD Biosciences, USA), 10,000 events were counted for each animal sample and the data was analyzed using CellQuest (BD Biosciences, USA) and WinMDI 2.9 software packages.

### Nitric oxide analysis

The production of NO was performed as described by Green *et al*. [31]. Supernatants were mixed in a ratio of 1:1 with the Griess reagent (1:1 volume 1% sulfanilamide in 5% phosphoric acid in deionized water with an equal volume of 0.1% N- [1-naphthyl]–ethylenediamine in deionized water). After 10 minutes, the mixture was read in an ELISA reader (540 nm) and quantification of NO production was based on a sodium nitrite standard curve.

### Cell Culture and viability assay

The mouse macrophage cell line J774.G8 were grown in plastic bottles in a RPMI 1640 medium (Sigma Chemical Company, St Louis, MO) supplemented with 10% fetal bovine serum (GIBCO-Life Technologies, Rockville, MD), penicillin (100 U/mL), streptomycin (100 μg/mL), glutamine (2 mM) and HEPES (15 mM; Biochrom AG) at 37°C in a humidified atmosphere of 5% CO_2_ (S1 Fig).

When cultures formed a confluent, monolayer cells were scrapped, centrifuged and put to adhere in 96 wells plate with RPMI at a density of 2 × 10^4^ cell/ml. The cultured cells were treated with 10, 20, and 40 μg/ml of the Açaí extracts for 24, 48 and 72 h. The supernatant was removed, and 10 μl of 3-(4,5-dimeth-ylthiazol-2-yl)-2,5-diphenyltetrazolium bromide (MTT) in RPMI medium was added to each well. Cells were incubated in a CO_2_ chamber for 3 h with protection from light. After the medium were aspirated, 100 μl of dimethyl sulfoxide (DMSO) was added to the cells to dissolve the formazan. The absorbance at 538 nm was measured with a Spectra Max 190 spectrophotometer (Molecular Devices, Sunnyvale, CA, EUA).

### Statistical analysis

Data are expressed as mean ± standard deviations (SD). Statistical comparisons between treated group and control were performed with Student t-test. For VEGF, VEGFR-2, MMP-9, COX-2 and F4-80 morphometric analysis, statistical calculations were carried out with use of the Stat-Xact-5 software program (CYTEL Software Corporation, Cambridge, MA). The relative quantification of the target genes was carried out using Delta-Delta Ct method. Cell culture and viability assay experiments were performed in triplicate (n = 3) and the data were expressed as mean ± SD. The Student-Newman-Keuls test was used to assess the presence of statistical differences between the groups when a statistically significant association was described by ANOVA. The level of significance for significant difference between groups was set at P <0.05 in all analyses.

## Results

### Açaí Suppresses Endometriosis Growth

**Fig 1:**
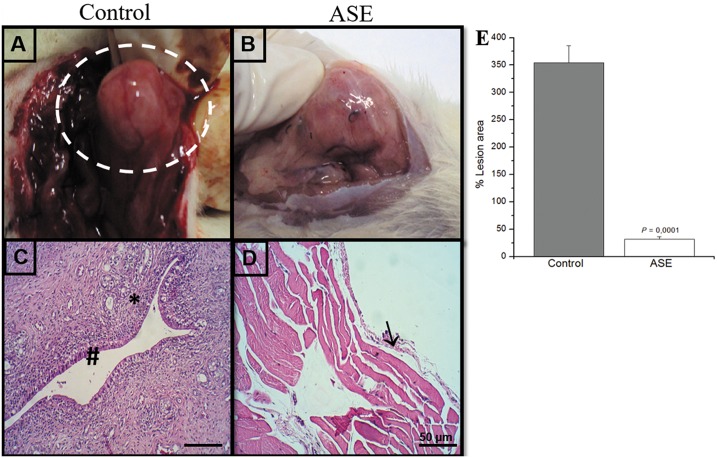
Morphological characteristics of rat peritoneal endometriotic lesions in control and açaí treated groups. In the control group (A), the lesions were cystic and vascularized and resembled human peritoneal endometriosis. In the açaí group (B), a drastic reduction in the growth of the lesions was visualized. Histologically, the control group (C) showed the presence of endometrial glands (#) and stroma cells (*), which confirmed the viability of lesions. In the açaí group (D), there was tissue atrophy and regression of lesions (→). Measurements of the lesion area demonstrated a statistically significant difference between the groups (E).

The endometrial explants formed viable cystic and well-vascularized lesions after 15 days in all 20 animals (Fig 1A). The histopathological results revealed typical endometrial components, such as glands and stroma, which confirmed the viability of the lesions (Fig 1C). The maintenance and growth of the lesions were suppressed in the treated animals group, and an important decrease in implant size was observed (Fig 1B). The histological analyses revealed atrophy and regression of the lesion areas (Fig 1D). Measurements of the lesions area confirmed these observations, which were significantly different between the two groups (Fig 1E). There was no significant difference in weight over time between the treated açaí group and the control group (data not shown).

### Açaí Inhibits Angiogenesis in Experimental Endometriosis

**Fig 2:**
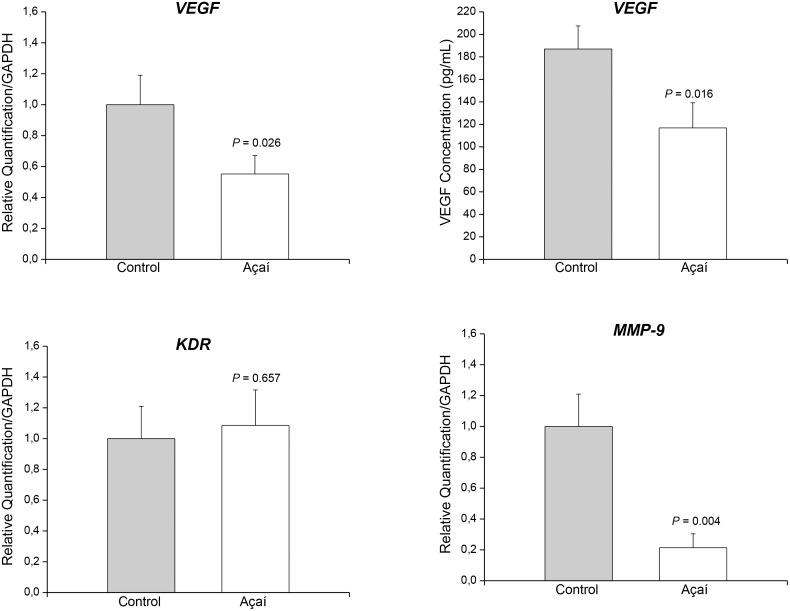
Açaí inhibits angiogenesis in experimental endometriosis. Expression of mRNA encoding for VEGF (A), KDR (C) and MMP-9 (D) assayed using RT-PCR. VEGF concentrations (B) were measured suing ELISA immunoassays in control endometriosis lesions and lesions treated with açaí. The levels of VEGF and MMP-9 mRNA transcripts and VEGF concentrations in the treated endometriotic lesions were significantly lower than the levels in the control lesions.

**Fig 3:**
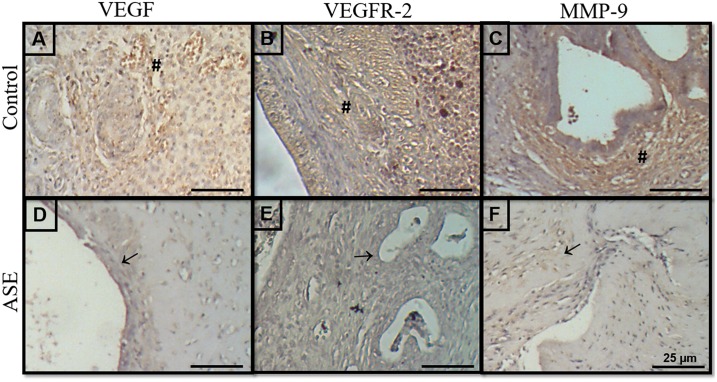
Açaí decreased angiogenesis markers immunodistribution in endometriosis. The immunoreactivity of VEGF, VEGFR-2 and MMP-9 was detected predominantly in the stroma (#), primarily around the glands in untreated endometriosis (A, B, C). Treated endometriotic lesions (D, E, F) exhibited a significant decrease in reaction intensity (→).

The angiogenesis process was investigated using mRNA expression, immunostaining and ELISA immunoassays based on MMP-9, VEGF and its receptor VEGFR-2 (S1 Table). Quantitative real-time PCR demonstrated suppression of the levels of VEGF (Fig 2A) and MMP-9 (Fig 2D) mRNA transcripts, and ELISA revealed a decrease in VEGF concentration (Fig 2B) in the endometriotic lesions treated compared to the control group. However, KDR mRNA transcripts were not different among the two groups (Fig 2C). VEGF, VEGFR-2 and MMP-9 immunoreactivity was detected in the lesions, predominantly in the stroma, around the glands, and in the cytoplasm of endothelial cells in non-treated endometriosis. The distribution of these three angiogenic markers decreased in endometriosis animals treated with açaí (Fig 3D, 3E and 3F) compared to the control group (Fig 3A, 3B and 3C). Histomorphometry evaluations of VEGF, VEGFR-2 and MMP-9 confirmed the observation of significant decreases (*P* = .0001) in these markers in endometriosis animals treated with açaí (2.5 ± 0.9, 2.8 ±0.6 and 4.2 ± 0.8, respectively) compared to the control group (22.1 ± 1.1, 25.4 ± 2.5 and 27.5 ± 2.4, respectively).

### Anti-inflammatory effect of açaí on endometriotic lesions

**Fig 4:**
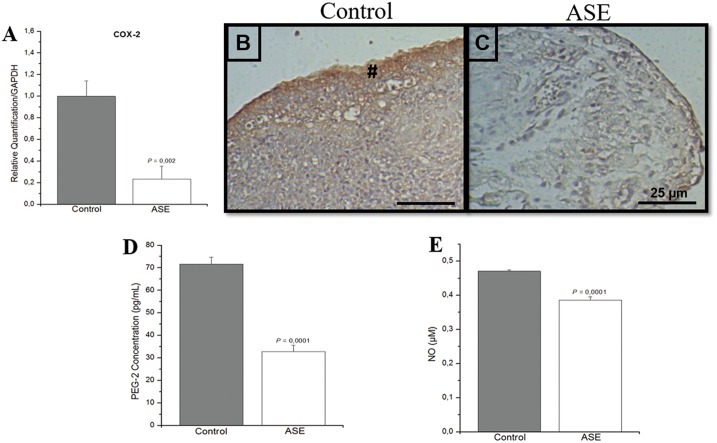
Anti-inflammatory effect of açaí on endometriotic lesions. COX-2 mRNA transcripts in the control group were higher than the açaí group (A). COX-2 immunoreactivity was detected predominantly in the glandular epithelial cells (#) in the control group (B) compared to lesions treated with açaí (C). PGE_2_ levels (D) and NO production (E) were higher in the control group than in the açaí group.

The inflammatory profile of endometriotic lesions was analyzed using COX-2 mRNA expression, COX-2 immunostaining, and an ELISA immunoassay of prostaglandin 2 (PGE_2_) (S2 Table). The level of COX-2 mRNA transcripts (Fig 4A) and the intensity of the COX-2 reaction (Fig 4C) were reduced in endometriotic lesions treated with açaí compared to the control group (Fig 4B). Histomorphometry evaluation of COX-2 was significantly smaller (*P* = .0001) in endometriosis animals treated with açaí (5.9 ± 0.5) compared to the control group (30.8 ± 1.7). PGE_2_ (Fig 4D) and NO (Fig 4E) concentrations were significantly higher in the control group compared to the açaí group, which demonstrated the anti-inflammatory potential of the extract.

### Açaí decreased levels of macrophages

**Fig 5:**
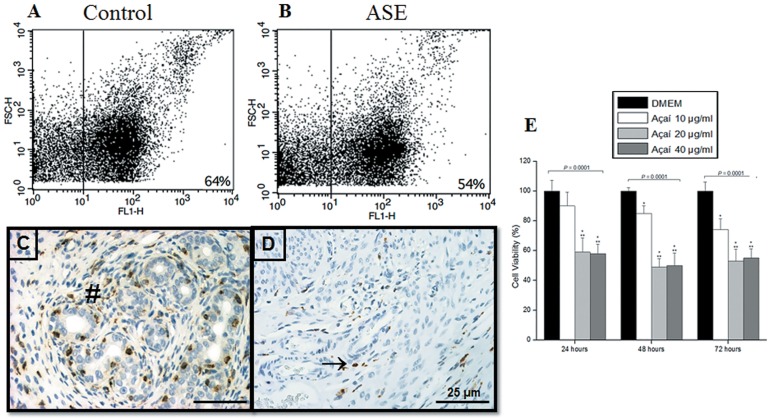
Açaí decreased levels of macrophages *in vivo* and *in vitro*. FACS analysis (A and B) of the macrophage phenotype (FL1-H) revealed fewer macrophages in the treated group than the control group population. F4-80 immunoreactivity (C and D) revealed that the higher number of positive macrophages in the stroma, primarily around the glands in control endometriosis (#) was drastically reduced in açaí treated lesions (→). Similarly, the MTT assay showed that the extract of açaí caused significant reduction in macrophage cell line viability after 24, 48 and 72 h of treatment (E). Values are mean ± standard deviations, and P = ANOVA test. *Significant difference compared to DMEM group and **significant difference compared to açaí 10 μg/ml group (Student-Newman-Keuls test, P < 0.05).

We analyzed the presence of F4/80-positive cells in the endometriotic lesions of both groups because of the role of macrophages in angiogenesis and inflammation. We identified a decrease in the number of macrophage-positive cells in treated endometriotic lesions (Fig 5B) compared to the control group (Fig 5A). We analyzed the presence of these cells in endometriotic tissue using immunostaining of a macrophage activation marker to confirm these observations. We observed an important decrease in the number of positive cells in the stroma compartment in the treated group (Fig 5D) compared to the control group (Fig 5C). The histological scores of F4-80 immunostaining (S3 Table) confirmed these results (control, 49.3 ± 2.1 versus açaí 15.3 ± 1.0, *P* = .0001). We also performed the viability assay using macrophage cell line J774.G8 treated with 10, 20 and 40 μg/ml of açaí after 24, 48 and 72 h, and observed the significantly decreased the viability of macrophages cells in all açaí-treated, except treatment with 10 μg/ml for 24 h (Fig 5D). Macrophages cells treated with 20 and 40 μg/ml of açaí reduced cell viability in about 50% after all times, reinforcing the hypotesis that the açaí acts directly in the macrophage decrease. In addition, we made hematological analyses and observed a marked lymphocytosis in control animals compared to the açaí group (S3 Table). A recovery of leukocyte numbers to normal parameters was observed in the açaí group compared to the group of animals without endometriosis (data not shown).

## Discussion

Endometriosis frequently produces serious effects on professional, social and marital life because it is associated with infertility and severe and incapacitating painful symptoms, including chronic pelvic pain, dysmenorrhea and dyspareunia [3,32]. Therefore, pharmacological treatments for endometriosis maintain a hypoestrogenic environment and relieve pelvic pain, but the side effects of these therapies limit their long-term use. Natural therapies were used recently for the treatment of various inflammatory diseases, and these therapies may be an effective treatment strategy for endometriosis [33,34].

Several studies demonstrated that açaí possesses high levels of phytochemicals with antioxidant, antinociceptive, anti-inflammatory, hypocholesterolemic, and anticancer activities [20–26,35,36]. To our knowledge, this report is the first study to evaluate the anti-inflammatory and antiangiogenic effects of açaí in endometriotic implants. Notably, our study used the hydro-alcoholic extracted from açaí stones because this extract was previously described to be more active than the pulp extract [25,37].

An important decrease in the implant size was observed in the animals treated with açaí, and the histopathological findings revealed marked atrophy and lesion regression. Other phytomedicines also suppressed endometriosis in a murine model. Ergenoglu *et al*. used the natural polyphenol resveratrol and demonstrated a significant reduction in implant size and considerable histological changes in endometrial foci at the end of the treatment period [38]. Another study examined the effects of a plant polyphenol extracted from the traditional Chinese medicine turmeric, curcumin, on endometriosis. Curcumin treatment decreased the ectopic endometrial glands and narrowed the lumen [39]. These studies support the potential application of phytomedicines as endometriosis treatments.

Angiogenesis is pivotally important in the development of endometriosis [40,41], and antiangiogenic agents from different substance groups are discussed as possible candidates for a new therapeutic approach [42–44]. Açaí treated animals in our study exhibited reduced VEGF expression, which is the most prominent pro-angiogenic factor in endometriosis. Similar results of reduced VEGF expression were reported in endometriosis models treated with a prodrug of green tea and epigallocatechin-3-gallate [45–48]. We also demonstrated that the açaí extract decreased VEGFR-2 distribution. These results are important because the binding of VEGF to VEGFR-2 enhances endothelial cell migration, proliferation and the release of various proteolytic enzymes [49].

Endometriosis is considered a benign disease, but it often presents characteristics of malignancy [40,50]. Women with endometriosis also exhibit an increased risk of cancer, especially ovarian cancer [50,51], which primarily occurs because both conditions require angiogenesis for maintenance and growth [52]. Some studies demonstrated an anti-tumorigenic activity of açaí in HL-60 leukemia cells and attenuation of colon carcinogenesis in rats [53,54]. Silva *et al*. demonstrated the anticancer activity of açaí in different malignant cell lines [25]. These authors concluded that the extract exhibited anti-tumorigenic potential in the human breast cancer MCF-7 cells and reduced viability and morphological alterations [25]. These results may be attributed to the rich content of polyphenols, anthocyanin and flavonoids in açaí because these substances possess anticancer activity [25,53,54].

The invasive properties of endometriotic tissue are also related to the increased proteolytic activity and matrix remodeling. MMPs are essential for the degradation of the extracellular matrix, which contributes to endometriosis development [55,56]. Our study demonstrated that açaí played an important role in reducing the MMP-9 levels in endometriotic lesions. Similar studies using curcumin also demonstrated reduced MMP expression in the intrinsic apoptotic pathway [57,58]. Some authors reported that the increase in ROS was a major factor that increased MMP expression and activity [59,60]. Costa *et al*. demonstrated that açaí treatment downregulated MMPs in experimental hypertension, and suggested that this downregulation was the result of the antioxidant activity of açaí [35].

High levels of ROS are generated in endometriosis and induce cell damage and proliferation [61], which supports the important role of peritoneal macrophages in the secretion of pro-inflammatory/proangiogenic cytokines [56,62]. Our findings demonstrated that the presence of F4/80-positive macrophages and NO concentrations were higher in endometriotic lesions and statistically reduced in animals treated with açaí. These results support the role of flavonoids as the major polyphenols in açaí that exhibit large anti-oxidant activity [19]. Several studies described the pivotal importance of macrophages in pathophysiology of endometriosis [4,8,40,56] and we also demonstrated that açaí decreased the viability of macrophages line cells *in vitro*. Therefore, we suggest that açaí decrease of macrophages hence reduced ROS production and decreased the inflammatory and angiogenesis processes in endometriotic lesions.

We also investigated COX-2 and PGE_2_ signals to elucidate the mechanisms of action of açaí in the endometriosis inflammatory process. Several authors previously described that PGE_2_ induced VEGF and played an important role in the pathogenesis of endometriosis [56,63,64]. We observed reduced COX-2 and PGE_2_ levels in animals treated with açaí, and this result was likely due to the high levels of anthocyanin in açaí [65]. Notably, the suppression of COX-2 expression by açaí may be a prerequisite for the effective control of endometriosis-related pain. This hypothesis is supported by the significant and potent antinociceptive effect of this extract in rodent models of acute and neuropathic pain [26].

Considerable evidence supports that a diet rich in polyphenols reduces the risk for many pathological conditions, including cardiovascular disease and cancer [66]. However, studies demonstrated an effect of fruits and vegetables on the risk of cancer, and many of the tested extracts have genotoxic potential [67,68]. Ribeiro *et al*. investigated the genotoxicity of açaí in bone marrow, peripheral blood, liver and kidney cells of mice using the micronuclei test and comet assay [69]. The results demonstrated that gavage administration of açaí was not genotoxic in mice, and its components may be exploited as a promoter of good health [69].

**Fig 6:**
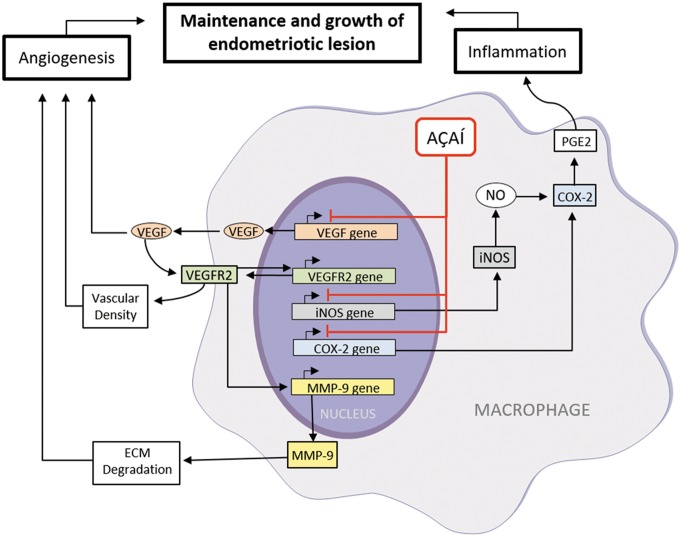
Açaí signaling pathways in endometriosis. In the endometriotic microenvironment, the macrophages are essential to promote the angiogenesis process and inflammation because it leads to increase the VEGF, iNOS and COX-2 genes expression. Açaí acts in this pathway and decreases the number of activated macrophages resulting in the reduction of the target genes expression, suppressing the maintenance and growth of endometriotic lesion.

Finally, based on the results of this study and the previous ones, we propose a mechanism for the therapeutic effects of açaí for endometriosis (Fig 6). Macrophages play a key role in inflammatory and angiogenesis of endometriosis. We know that the macrophages are an important source of VEGF and it is essential in the establishment and growth of endometriotic lesion [4,40,70]. High concentration of VEGF returns to activated macrophage and bind with VEGFR-2. This signaling upregulating the MMP-9 transcription induces the extracellular matrix remodeling promoting the increase of the vascular density [71]. Moreover, the activated macrophages synthesize high concentration of inducible nitric oxide synthase (iNOS) and generate NO [61]. On the other hand, this process increased the COX-2 and PGE2 levels to enhance the inflammatory process and angiogenesis [56,63,64]. Therefore, we propose that açaí decreases the number of activated macrophages resulting in the reduction of the target genes expression, such as VEGF, iNOS and COX-2 suppressing the maintenance and growth of endometriotic lesion.

## Conclusions

In conclusion, the results of this study demonstrated the antiangiogenic and anti-inflammatory potential of açaí, which produced morphological alterations in endometriotic lesions. Açaí may also modulate the progress of endometriosis and suppress the symptoms related to pain, which supports the possible development of a novel and effective drug. The actual mechanisms of the beneficial effects of açaí on endometriotic lesions are not completely understood, but we are optimistic that these effects will be reproducible in clinical tests, and we will continue our research of this extract.

## Supporting Information

S1 Fig Macrophage cell line. Morphology observations by phase-contrast microscopy of macrophage cell line J774.G8 in plastic bottles cultivated in a RPMI 1640 medium (A and B).
(TIF)

S1 Table Immunostaining and ELISA immunoassays based on MMP-9, VEGF and its receptor VEGFR-2. (XLS)

S2 Table COX-2 immunostaining, and an ELISA immunoassay of PGE_2_. (XLS)

S3 Table F4-80 immunostaining, and hematological analyses. (XLS)
