# Supplementary figures and images for: Euterpe oleracea Extract (Açaí) Is a Promising Novel Pharmacological Therapeutic Treatment for Experimental Endometriosis
Source: PLoS One. 2016 Nov 16;11(11):e0166059. doi: 10.1371/journal.pone.0166059 (PMC5113045; doi:10.1371/journal.pone.0166059)

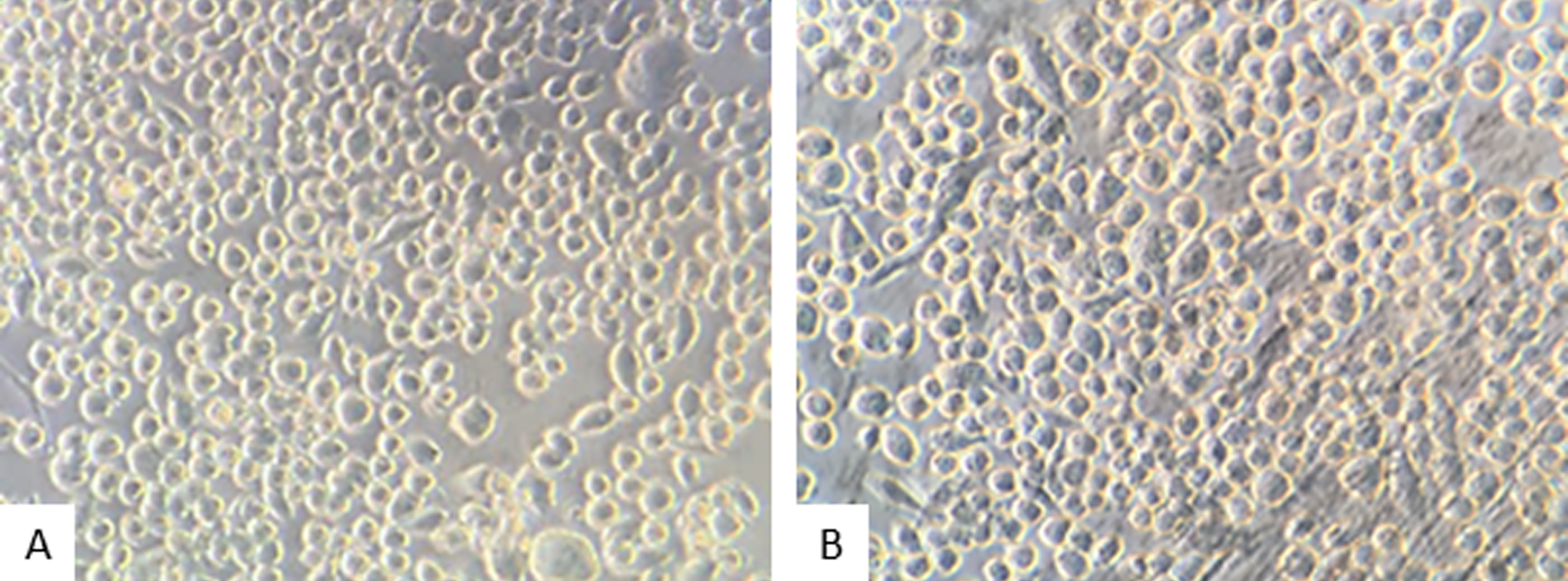

Supplement: S1 Fig — Morphology observations by phase-contrast microscopy of macrophage cell line J774.G8 in plastic bottles cultivated in a RPMI 1640 medium (A and B). (TIF) [file pone.0166059.s001.tif]
